# Mutant alleles of *FAD2-1A *and *FAD2-1B *combine to produce soybeans with the high oleic acid seed oil trait

**DOI:** 10.1186/1471-2229-10-195

**Published:** 2010-09-09

**Authors:** Anh-Tung Pham, Jeong-Dong Lee, J Grover Shannon, Kristin D Bilyeu

**Affiliations:** 1 University of Missouri, Division of Plant Sciences, 110 Waters Hall, Columbia, MO 65211 USA; 2 Division of Plant Biosciences, Kyungpook National University, Daegu 702-701, Republic of Korea; 3 University of Missouri, Division of Plant Sciences, University of Missouri-Delta Research Center, Portageville, MO 63873 USA; 4 USDA-ARS, Plant Genetics Research Unit, 110 Waters Hall, Columbia, MO 65211 USA

## Abstract

**Background:**

The alteration of fatty acid profiles in soybean [*Glycine max *(L.) Merr.] to improve soybean oil quality is an important and evolving theme in soybean research to meet nutritional needs and industrial criteria in the modern market. Soybean oil with elevated oleic acid is desirable because this monounsaturated fatty acid improves the nutrition and oxidative stability of the oil. Commodity soybean oil typically contains 20% oleic acid and the target for high oleic acid soybean oil is approximately 80% of the oil; previous conventional plant breeding research to raise the oleic acid level to just 50-60% of the oil was hindered by the genetic complexity and environmental instability of the trait. The objective of this work was to create the high oleic acid trait in soybeans by identifying and combining mutations in two delta-twelve fatty acid desaturase genes, *FAD2-1A *and *FAD2-1B*.

**Results:**

Three polymorphisms found in the *FAD2-1B *alleles of two soybean lines resulted in missense mutations. For each of the two soybean lines, there was one unique amino acid change within a highly conserved region of the protein. The mutant *FAD2-1B *alleles were associated with an increase in oleic acid levels, although the *FAD2-1B *mutant alleles alone were not capable of producing a high oleic acid phenotype. When existing *FAD2-1A *mutations were combined with the novel mutant *FAD2-1B *alleles, a high oleic acid phenotype was recovered only for those lines which were homozygous for both of the mutant alleles.

**Conclusions:**

We were able to produce conventional soybean lines with 80% oleic acid in the oil in two different ways, each requiring the contribution of only two genes. The high oleic acid soybean germplasm developed contained a desirable fatty acid profile, and it was stable in two production environments. The presumed causative sequence polymorphisms in the *FAD2-1B *alleles were developed into highly efficient molecular markers for tracking the mutant alleles. The resources described here for the creation of high oleic acid soybeans provide a framework to efficiently develop soybean varieties to meet changing market demands.

## Background

Soybean oil consumed in the U.S. accounted for approximately 70% of the total edible fat and oil consumption in 2008; three quarters of the soybean oil was used as cooking oil and baking and frying fat (http://www.soystats.com/2009/). Soybean oil's utilization is determined by its fatty acid composition, with commodity soybean oil typically containing 13% palmitic acid (16:0), 4% stearic acid (18:0), 20% oleic acid (18:1), 55% linoleic acid (18:2), and 8% linolenic acid (18:3). Consumption of oils with high oleic acid content is desirable because this monounsaturated fatty acid not only improves shelf life but also reduces the need for hydrogenation, a process adding to the cost of the oil and generating unwanted trans-fat that has been linked to many health problems in humans [1]. Additionally, in biodiesel production, there is also a need for oils high in oleic acid and low in saturated fatty acids in order to improve the oxidative stability while augmenting cold flow [2]. Moreover, the enhanced oxidative stability of soybean oil with higher oleic acid content will also open up a variety of food uses and industrial applications like spraying oils or machine lubricants [3].

In the lipid biosynthetic pathway, conversion of oleic acid (18:1) precursors to linoleic acid (18:2) precursors is catalysed by the delta-twelve fatty acid desaturase 2 enzyme (FAD2) [4,5]. While in *Arabidopsis *and maize only one single copy of a *FAD2 *gene was identified [4,6], soybean appears to possess multiple copies of the gene due to the consequence of repeated rounds of genome duplication followed by limited sequence loss [5]. The soybean *FAD2 *gene family has been previously characterized at the genome level for structure and expression [5]. Among the *FAD2 *genes identified in soybean, the FAD2-2 desaturases consisting of *FAD2-2A *(Glyma19g32930), *FAD2-2B *(Glyma19g32940), and *FAD2-2C *(Glyma03g30070) were found to be widely expressed in the vegetative tissues of the soybean plant [5]. The exception was *FAD2-2A*, for which expression was not detected; *FAD2-2A *was predicted to be nonfunctional as it has a deletion of 100 bp in the coding region [5]. The two microsomal FAD2-1 desaturases *FAD2-1A *(Glyma10g42470) and *FAD2-1B *(Glyma20g24530) were mainly expressed in developing seeds [5,7]. Thus, *FAD2-1A *and *FAD2-1B *are considered to play an important role in controlling the oleic acid level in developing soybean seeds and were selected as candidate genes to elucidate the molecular genetic basis of soybean lines from the germplasm collection that contained elevated levels of oleic acid.

*FAD2-1A *and *FAD2-1B *are most closely related to one another, with a shared genomic organization containing a single intron and 99% identity in encoded amino acid sequence, and are present on homologous chromosome regions mapped to linkage group O (chromosome 10) and I (chromosome 20), respectively [5,7]. Characterization of the expression of the individual soybean *FAD2 *genes confirmed the importance of *FAD2-1A *and *FAD2-1B *with expression of these genes during peak oil synthesis; a possible role was also revealed for *FAD2-2C *under cool temperature conditions [5]. Differential response to temperature was also demonstrated for the soybean FAD2-1A and FAD2-1B enzymes expressed in yeast [7]. The temperature during soybean pod fill has been shown to influence the fatty acid composition of soybean oil, with cooler temperatures leading to increased oleic acid desaturation and decreased oleic acid accumulation [8-10].

Selection for breeding and genetic engineering resulting in elevated oleic acid levels were reported in many oilseed crops: safflower[11], sunflower [12], peanut [13-15], canola [16,17], cotton [12] and maize [6]. While the elevated oleic acid phenotype was often observed after a single *FAD2 *gene was mutated, the very highest oleic acid phenotypes (> 80% of the total oil) were achieved most frequently by silencing all copies of *FAD2 *genes that were expressed in developing seeds, or in particular for peanut, by combining the mutation in the active site of *ah*FAD2A with the loss of transcription of *ahFAD2B *[14,15]. No soybean lines exist in the USDA National Plant Germplasm System collection with the high oleic acid trait (oleic acid content above 70% of the oil fraction), although multiple lines contain elevated oleic acid levels [9].

Several elevated oleic acid soybean lines have been characterized at the molecular level. The destruction of the *FAD2-1A *gene by X-ray mutagenesis yielded two soybean lines with oleic acid content of approximately 50% of the oil [18-20]. A reverse genetics approach was utilized to identify a soybean line containing a missense mutation in *FAD2-1A *that associated with an elevated oleic acid content of the oil [21]. Many soybean lines were developed through recurrent selection that contained elevated oleic acid content such as N00-3350, N98-4445A, and N97-3363-3 [22], but the genetic basis for the trait was extremely complex, with at least six QTLs conditioning the phenotype [23,24]. In addition, the level of oleic acid in the oil of these soybean lines was particularly susceptible to environmental effects when compared to the X-ray *FAD2-1A *deletion line [9,10]. No mutations in *FAD2-1B *that associate with elevated oleic acid content have been reported to date.

Suppression of *FAD2-1 *gene expression by means of genetic engineering has been successful in creating soybean lines with the high oleic acid trait, with oleic acid content above 80% of the oil fraction reported and very little environmental impact on the trait [3,25,26]. Transgenic expression of ribozyme terminated sense or antisense *FAD2-1 *constructs was successful in eliminating the *FAD2-1 *mRNA expression signal in developing embryos and producing siRNAs for the *FAD2-1 *genes [25]. No attempt was made to distinguish between the *FAD2-1A *and *FAD2-1B *genes in the transgenic work, and it is also possible that the *FAD2-2 *genes were targeted in the experiments [25].

The objective of this work was to create the high oleic acid trait in soybeans using conventional plant breeding technology. We hypothesized that combinations of mutant alleles of the soybean *FAD2-1A *and *FAD2-1B *genes would greatly reduce the FAD2 enzyme activity in developing seeds and thus result in an accumulation of oleic acid at the expense of linoleic and linolenic acid in the triacylglycerol fraction of the seed oil. We took a candidate gene approach with the *FAD2-1A *and *FAD2-1B *genes present in soybean germplasm accessions containing elevated levels of oleic acid in the oil. Two mutant alleles of *FAD2-1B *were identified that associated with elevated oleic acid content. In addition, mutant alleles of *FAD2-1A *and *FAD2-1B *were combined to create soybeans with the high oleic acid trait.

## Results

### Identification of mutant alleles of *FAD2-1B *in soybean lines PI 283327 and PI 567189A

**Figure 1:**
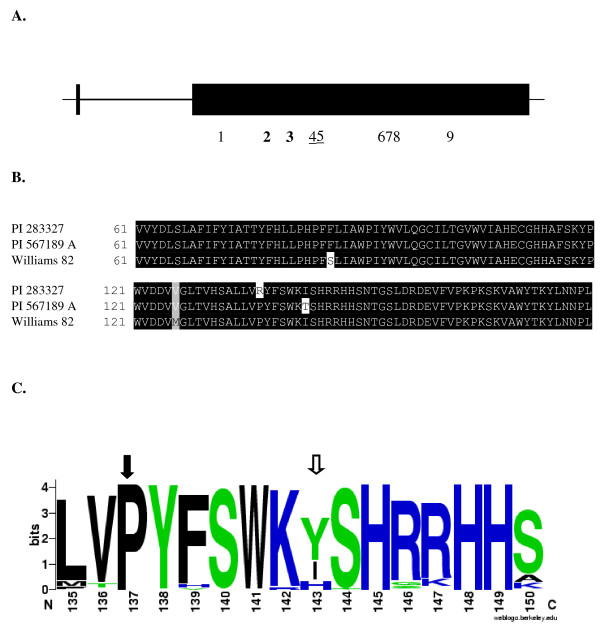
**Characterization of mutations in the *FAD2-1B *alleles from soybean lines PI 283327 and PI 567189A**. **A**. Schematic of the *FAD2-1B *gene and the polymorphisms identified in the alleles from PI 283327 and PI 567189A. The horizontal line represents the DNA for *FAD2-1B*, with the heavier line indicating the intron and lighter lines representing the 5' and 3' untranslated portions of the gene. The dark vertical line represents the portion of exon 1 that contains only the start codon and the darkened rectangle represents exon 2. Numbers beneath the schematic indicate the positions of polymorphisms compared to the Williams 82 reference *FAD2-1B *coding sequence, with shared missense mutations in bold and non-shared missense mutations underlined. 1 = a105g, silent; 2 = c257t, S86F; 3 = a376g, M126V; 4 = c410g, P137R unique to PI 283327 and PI 210179; 5 = t428c, I143T unique to PI 567189A and PI 578451; 6 = c657t, silent; 7 = t669c, silent; 8 = t682c, silent; 9 = a918g, silent. **B**. Fatty acid desaturase FAD2-1B amino acid sequence alignment in the region surrounding the mutations in PI 283327 and PI 567189 A. Amino acid positions are indicated at the beginning of each line of the alignment. Identical amino acid residues are highlighted in black, a similar amino acid substitution is highlighted in gray, and the S86F, P137R, and I143T mutations are not highlighted. **C**. Weblogo output of the amino acid conservation FAD2 enzyme as part of the BLINK feature at NCBI using GI number 197111722. The top 100 best matched sequences were aligned and used as input for sequence LOGO http://weblogo.berkeley.edu/logo.cgi. The logo consists of stacks of symbols, one stack for each position in the amino acid sequence. The overall height of the stack indicates the sequence conservation at that position, while the height of symbols within the stack indicates the relative frequency of each amino acid at that position. Closed arrow indicates residue changed due to the P137R *FAD2-1B *mutation in PI 283327 and open arrow indicates residue changed due to the I143T *FAD2-1B *mutation in PI 567189A.

In an effort to identify novel alleles of the soybean *FAD2-1A *and *FAD2-1B *genes, genomic DNA was characterized for the sequence of both genes from plant introduction (PI) lines selected from the National Genetic Resources Program containing elevated oleic acid levels from 27 to approximately 50% percent of the oil, while commodity soybeans typically produce 19-23% oleic acid [27]. The *FAD2-1A *alleles from PI 283327 and PI 567189 A were identical to the reference 'Williams 82' [28] allele. In contrast, for the *FAD2-1B *genes from PI 283327 and PI 567189 A, seven common single nucelotide polymorphisms (SNPs) and one unique SNP for each line were identified when compared to the reference sequences from the cultivar 'Williams 82' (Figure 1A). Other soybean lines were also characterized for their *FAD2-1B *alleles, and two independent lines, PI 210179 and PI 578451, contained exactly the same *FAD2-1B *alleles as PI 283327 and PI 567189 A, respectively.

For the PI 283327 *FAD2-1B *allele, there were three missense mutations resulting in S86F, M126V, and P137R (Figure 1B). For the PI 567189 A *FAD2-1B *allele, two of the missense mutations were identical (S86F and M126V), and the third missense mutation was unique, I143T. Other soybean lines were identified that had *FAD2-1B *alleles which contained different combinations of the silent mutations and the S86F and M126V missense mutations (data not shown).

To predict the potential effect of the amino acid changes to soybean FAD2-1B enzyme function, we used the program PolyPhen to analyze the potential severity of each amino acid change [29]. In addition, the relative amino acid conservation for each position in the enzyme was evaluated visually using Weblogo after alignment of 100 different FAD2 protein sequences present in the National Center for Biotechnology Information database [30]. The two shared amino acid substitutions M126V and S86F each occur at a highly variable position according to the alignment in Weblogo and were classified by Polyphen as benign substitutions, indicating they are likely to have no phenotypic effect. For the M126V change, methionine and valine have similar chemical properties.

*FAD2-1B *alleles that encoded only the S86F misssense mutation were identified and analyzed for the functional consequence of that isolated mutation. Although serine and phenylalanine are amino acids with different chemical properties, several lines of evidence indicated that the S86F *FAD2-1B *allele was functional, including detection of delta-twelve fatty acid desaturase activity in a yeast recombinant expression experiment and no association of the S86F *FAD2-1B *allele variant with an elevated oleic acid phenotype (data not shown).

The *FAD2-1B *P137R mutation present in PI 283327 represents a charge change for the substituted amino acid since proline is nonpolar while arginine is basic. At position 137 of wild-type FAD2-1B, the proline is perfectly conserved; a proline at that position is invariant for all of the tested FAD2 sequences represented in the protein database (Figure 1C). In PolyPhen, the amino acid change was classified as probably damaging for P137R, which means that this SNP is predicted to affect protein structure and/or function.

For the isoleucine present at position 143 of wild-type FAD2-1B, other amino acids were observed in this position in the FAD2 sequences present in the protein database, indicating that an isoleucine at position 143 is less conserved than the proline at the 137 position (Figure 1C). However, the substitution of threonine for isoleucine at this position was not observed in the database analysis. Moreover, isoleucine and threonine also have contrasting chemical properties since isoleucine is nonpolar while threonine is uncharged polar. In PolyPhen, the I143T amino acid change was classified as probably damaging. The conservation of amino acids in the general region of the *FAD2-1B *P137R and I143T mutations combined with the chemical nature of the changes is suggestive of the potential deleterious effects of these mutations to the FAD2-1B enzyme's structure and function.

### The PI 283327 *FAD2-1B *allele is associated with an increase in seed oleic acid content

**Figure 2:**
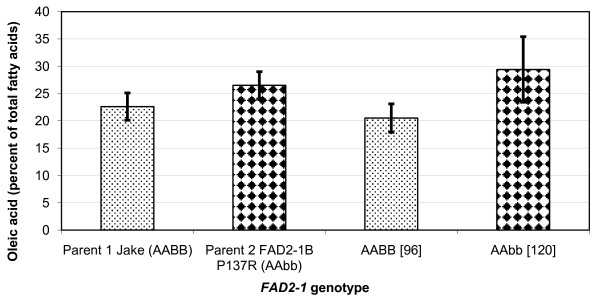
**Seed oleic acid phenotype and *FAD2-1 *genotype association analysis for population 1**. Average oleic acid content of soybean seeds produced in Portageville, MO from the F_6 _RILs developed from the cross Jake × PI 283327, population 1. Labels on the X-axis include: two parents with contrasting *FAD2-1B *genotypes and the RILs grouped by *FAD2-1B *genotype. BB indictates wild-type *FAD2-1B *alleles, and bb indicates mutant P137R *FAD2-1B *alleles derived from PI 283327; brackets surround the number of soybean seed samples represented in the genotype class. Error bars indicate plus and minus one standard deviation from the mean. Oleic acid phenotype data of each genotype class is the mean of oleic acid content as a percentage of the total fatty acid content of the oil of all lines carrying the *FAD2-1 *genotype, four individual samples for each line.

To test the hypothesis that the newly identified mutations in *FAD2-1B *are causative for the elevated oleic acid level in the plant introduction lines, an analysis of the oleic acid phenotype and *FAD2-1B *genotype association was examined for Population 1, an F_6 _recombinant inbred line (RIL) population developed from the cross 'Jake'[31] × PI 283327. The commodity soybean line Jake typically produces approximately 22% oleic acid in the seed oil and contains functional *FAD2-1A *and *FAD2-1B *alleles, represented as genotype *FAD2-1 *AABB. PI 283327 was selected from the germplasm collection because of elevated levels of oleic acid in the seed oil and carries the mutant *FAD2-1B *P137R allele, represented as genotype *FAD2-1 *AAbb with the lowercase allele designation always specifying the mutant allele and the capital case specifying the wild-type allele. Of the 54 lines in the RIL population 1 that contained homozygous alleles of *FAD2-1B*, the 30 lines carrying the mutant *FAD2-1B *P137R alleles from PI 283327 had an average of 29.4% oleic acid, while 24 lines carrying wild type alleles had an average of 20.5% oleic acid (Figure 2). Although the variation in the data was large, the difference in oleic acid contents between the two contrasting *FAD2-1B *genotypes was confirmed significant using Student's t-test at the 0.05 probability level (P > 0.05).

### Combinations of mutations in *FAD2-1A *and *FAD2-1B *produce high oleic acid levels in the seed oil

We hypothesized that combining the mutant alleles of both *FAD2-1A *and *FAD2-1B *in one soybean line would eliminate most of the enzyme activity responsible for converting oleic acid precursors into linoleic acid precursors, and thus result in a higher oleic acid content compared to soybean lines containing mutations in either gene individually. Two mutations in *FAD2-1A *were available: the deletion of the *FAD2-1A *gene in soybean line M23 (designated herein as *FAD2-1A *Δ) [18] and the missense mutation in *FAD2-1A *from line 17D (designated herein as *FAD2-1A *S117N) [21]. The identification of the two missense mutant alleles in *FAD2-1B *from PI 283327 (*FAD2-1B *P137R) and PI 567189A (*FAD2-1B *I143T) created the opportunity to evaluate the oleic acid phenotype in soybean lines containing different combinations of mutant *FAD2-1A *and *FAD2-1B *alleles.

**Figure 3:**
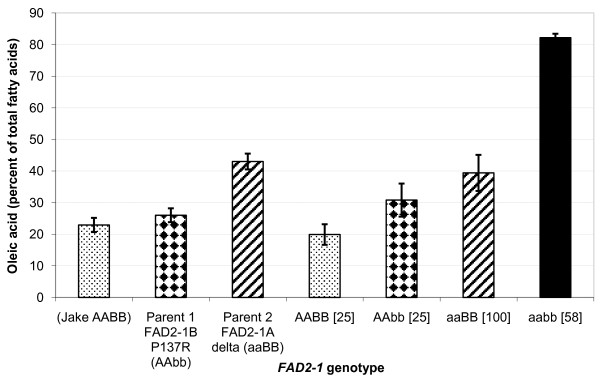
**Seed oleic acid phenotype and *FAD2-1 *genotype association analysis for population 2**. Average oleic acid content of soybean seeds produced in Portageville, MO from the segregating F_6 _and F_7 _RILs developed from the cross M23 × PI 283327, population 2. X-axis labels indicate a typical commodity soybean line (Jake), the two RIL parents, and the RILs grouped by *FAD2-1A *and *FAD2-1B *genotype. AA = wild-type *FAD2-1A *alleles, aa = mutant *FAD2-1A*Δ alleles derived from M23, BB = wild-type *FAD2-1B *alleles, bb = mutant P137R *FAD2-1B *alleles derived from PI 283327; brackets surround the number of soybean seed samples represented in the genotype class. Error bars indicate plus and minus one standard deviation from the mean. Oleic acid phenotype data of each genotype class is the mean of oleic acid content as a percentage of the total fatty acid content of the oil of all lines carrying the *FAD2-1 *genotype, five individual samples for each line, with two exceptions, one for class aaBB, where six lines had ten seed samples and eight lines had five seed samples. Additionally, two separate individual seed samples from the aabb lines were ommitted from the analysis because their genotypes could not be verified. Including these two samples would have led to a mean of 80.8 ± 8.0% oleic acid for the *FAD2-1 *aabb genotype class. Subsequent sampling of ten additional seeds from these lines produced oleic acid levels with a range of 81.5% to 86.2% oleic acid in the seed oil.

An association analysis of the oleic acid phenotype and the *FAD2-1A *and *FAD2-1B *genotypes was performed for Population 2, a RIL population consisting of F_2:6 _and F_2:7 _lines developed from the cross M23 × PI 283327 and grown in Portageville MO in 2008. Since soybean line M23 contained *FAD2-1A *Δ and wild-type alleles of *FAD2-1B*, the genotype is herein represented as *FAD2-1 *aaBB with the lowercase allele designation always specifying the mutant allele and the capital case specifying the wild-type allele; likewise, the *FAD2-1 *P137R genotype of PI 283327 is represented here as *FAD2-1 *AAbb. Individual seeds from each of 40 lines produced in an appropriate field environment and carrying the different homozygous combinations of *FAD2-1A *and *FAD2-1B *were analyzed along with the parental lines for the fatty acid phenotype of the seed oil (Figure 3). Transgressive segregation for oleic acid content was observed for the lines that inherited the *FAD2-1 *AABB and aabb genotypes, while the lines that recovered the parental *FAD2-1 *genotypes contained oleic acid contents similar to the phenotype of the parental lines. Lines with the genotype *FAD2-1 *AABB had an average oleic acid content similar to that of a conventional soybean 'Jake', which was 22.6% of total oil content. In contrast, individuals with the genotype *FAD2-1 *aabb had an average of 82.2% oleic acid, with a very narrow standard deviation of 1.2%. In this population, lines with either homozygous mutant *FAD2-1A *or *FAD2-1B *alleles had an average of 39.4% and 30.6% oleic acid, respectively, reiterating the relatively minor increase in oleic acid level conditioned by the *FAD2-1B *alleles in the presence of functional *FAD2-1A *alleles.

**Figure 4:**
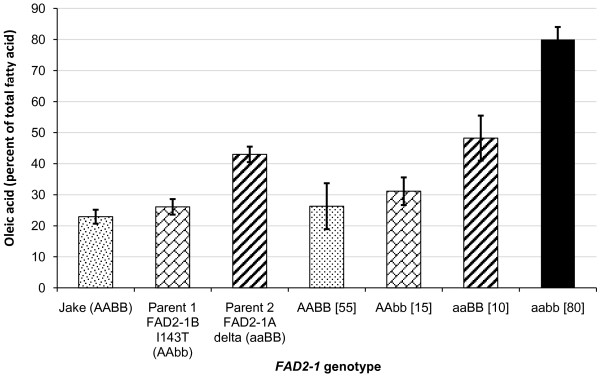
**Seed oleic acid phenotype and *FAD2-1 *genotype association analysis for population 3**. Average oleic acid content of soybean seeds produced in Costa Rica from the segregating F_6 _and F_7 _RIL developed from the cross M23 × PI 567189 A population 3. X-axis labels indicate a typical commodity soybean line (Jake), the two RIL parents, and the RILs grouped by *FAD2-1A *and *FAD2-1B *genotype. AA = wild-type *FAD2-1A *alleles, aa = mutant *FAD2-1A*Δ alleles derived from M23, BB = wild-type *FAD2-1B *alleles, bb = mutant I143T *FAD2-1B *alleles derived from PI 567189 A; brackets surround the number of soybean seed samples represented in the genotype class. Error bars indicate plus and minus one standard deviation from the mean. Oleic acid phenotype data of each genotype class is the mean of oleic acid content as a percentage of the total fatty acid content of the oil of all lines carrying the *FAD2-1 *genotype, five individual samples for each line, with the exception of the aaBB class, for which there was only one line and ten individual seeds were analyzed.

A similar experiment investigated the impact on seed oleic acid levels for the genetic combination of the *FAD2-1A *Δ alleles from M23 with the alternate *FAD2-1B *I143T alleles present in PI 567189 A. For Population 3, a M23 × PI 567189A RIL population, we genotyped lines based on the fatty acid profiles of F_5 _seeds harvested in Costa Rica, and then selected to present here the 31 lines classified into four homozygous combinations of *FAD2-1A *and *FAD2-1B*. The average oleic acid contents of the four *FAD2-1 *genotypes were significantly different from each other and again demonstrated transgressive segregation (Figure 4). Lines with *FAD2-1 *genotype AABB had an average oleic acid content not statistically different from that of Jake. In contrast, soybean lines with the *FAD2-1*aabb genotype had an average of 80.3% oleic acid. The lines that recovered the parental *FAD2-1 *genotypes also recovered the respective parental oleic acid phenotype.

**Figure 5:**
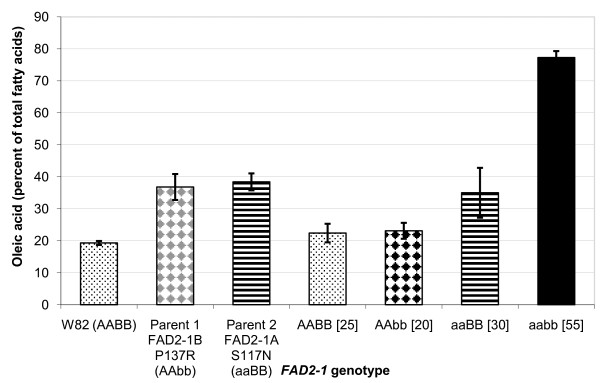
**Seed oleic acid phenotype and *FAD2-1 *genotype association analysis for F_3 _seeds of population 4**. Average oleic acid content of soybean seeds produced in Columbia, MO from the segregating F_2 _population developed from the cross 17D × S08-14788, population 4. X-axis labels indicate a typical commodity soybean line (Williams 82, W82), the two RIL parents, and the RILs grouped by *FAD2-1A *and *FAD2-1B *genotype. AA = wild-type *FAD2-1A *alleles, aa = mutant S117N *FAD2-1A *alleles derived from 17D, BB = wild-type *FAD2-1B *alleles, bb = mutant P137R *FAD2-1B *alleles derived from PI 283327; brackets surround the number of soybean seed samples represented in the genotype class. Error bars indicate plus and minus one standard deviation from the mean. Oleic acid phenotype data of each genotype class is the mean of oleic acid content as a percentage of the total fatty acid content of the oil of all lines carrying the *FAD2-1 *genotype, five individual samples for each line.

Soybean line 17D was discovered in a reverse genetics screen for mutations in *FAD2-1A *[21]. Line 17D contains elevated oleic acid in the seed oil due to a FAD2-1A missense mutation in a conserved amino acid referred to here as *FAD2-1A *S117N. Soybean lines which contained the *FAD2-1A *S117N alleles consistently accumulated lower oleic acid levels in the seed oil than lines containing *FAD2-1A *Δ alleles derived from M23, and the phenotype was not stable in different environments [21]. We next examined the combining ability in Population 4, the F_2 _and F_2:3 _individuals from the cross of the *FAD2-1A *S117N alleles derived from line 17D with the *FAD2-1B *P137R alleles derived from PI 283327 (17D × S08-14788). Homozygous *FAD2-1A *and *FAD2-1B *allele combinations were selected from *FAD2-1 *genotyped F_2 _plants for field growth in an appropriate environment and subsequent F_3 _seed oil fatty acid phenotype determination (Figure 5). Transgressive segregation was observed for the genotypes that inherited both the homozygous wild-type *FAD2-1 *alleles and the homozygous mutant *FAD2-1 *allele combinations. The *FAD2-1 *aabb combination demonstrated an average oleic acid content of 77.3%; the AABB combination displayed a typical commodity soybean oleic acid level. The parental oleic acid phenotype was recovered for the *FAD2-1 *aaBB genotype but not for the *FAD2-1 *AAbb genotype.

### Excess desaturase activity: A single wild-type *FAD2-1 *allele prevents high oleic acid accumulation

**Figure 6:**
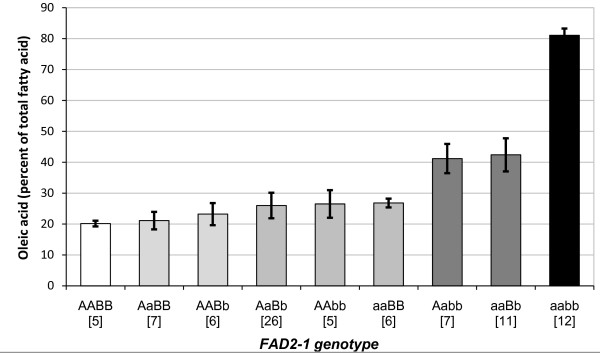
**Seed oleic acid phenotype and *FAD2-1 *genotype association analysis for F_2 _seeds of population 4**. Average oleic acid content of F_2 _soybean seeds produced in Costa Rica from the segregating population developed from the cross 17D × S08-14788, population 4. X-axis labels indicate a the soybeans grouped by *FAD2-1A *and *FAD2-1B *genotype. AA = wild-type *FAD2-1A *alleles, aa = mutant S117N *FAD2-1A *alleles derived from 17D, BB = wild-type *FAD2-1B *alleles, bb = mutant P137R *FAD2-1B *alleles derived from PI 283327; brackets surround the number of soybean seed samples represented in the genotype class. Error bars indicate plus and minus one standard deviation from the mean. Oleic acid phenotype data of each genotype class is the mean of oleic acid content as a percentage of the total fatty acid content of the oil of all lines carrying the *FAD2-1 *genotype.

Our initial investigation of both the *FAD2-1 *genotype and fatty acid phenotype in F_2 _seeds from Population 4 (*FAD2-1A *S117N × *FAD2-1B *P137 cross) demonstrated the epistatic nature of the mutant alleles working in combination, and the results revealed that only homozygous combinations of both mutant *FAD2-1A *and *FAD2-1B *were capable of producing the high oleic acid phenotype. Of the 200 F_2 _seeds that were phenotyped, there were 12 individual F_2 _seeds with genotype *FAD2-1 *aabb, and they had an average oleic acid content of 81%, ranging from 75.2% to 83.9% oleic acid (Figure 6). The next highest oleic acid phenotype in the set was 48.8%, and that seed had the *FAD2-1 *Aabb genotype. For a two recessive gene model, one sixteenth of the individuals should inherit the phenotype; recovery of 12 individuals with the high oleic acid phenotype satisfies this expectation by Chi-Square test at the 0.05 probability level.

Individuals with a single wild-type version of either *FAD2-1A *or *FAD2-1B *in combination with three mutant *FAD2-1 *alleles (Aabb or aaBb) contained approximately 40% oleic acid. No seeds from any of the other *FAD2-1 *genotypes contained oleic acid levels above 49% of the seed oil. Individuals with two or more wild-type *FAD2-1 *alleles contained oleic acid content with a range of 18-47% of the seed oil.

The necessity of the homozygous *FAD2-1A *and *FAD2-1B *mutant combination requirement for the high oleic acid phenotype was confirmed in an independent analysis of *FAD2-1 *genotype and fatty acid phenotype of field produced F_2 _seeds that contained homozygous *FAD2-1A *Δ alleles but which were segregating for *FAD2-1B *P137R alleles (Population 5). While the average oleic acid level of those seeds with the *FAD2-1 *aabb genotype was 82.5%, *FAD2-1 *aaBb seeds averaged 55.4%; *FAD2-1 *aaBB seeds averaged 43.4% oleic acid in the seed oil. The presence of a single wild-type version of the *FAD2-1B *allele also prevented a high oleic acid content in the seed oil, although the magnitude of the difference was greater for the F_2 _seeds from Population 4.

### The high oleic acid phenotype is stable in plants grown in alternate environments

Some of the high oleic acid soybean lines developed in this study demonstrated stability for the high oleic acid trait when grown in different environments (Table 1). Of the three environments, Costa Rica typically has the warmest temperatures during seed development, followed by the Portageville, MO environment; the Columbia, MO environment is the coolest of the three environments during seed development [9,10]. The differences in the oleic acid contents between environments when the *FAD2-1B *P137R alleles were present were minor. Soybean lines with genotype *FAD2-1*aabb of population 2 and 4 produced more than 80% oleic acid content in Costa Rica and Portageville, MO environments, and the oleic acid level was an average of 2-4% lower when grown in the Columbia, MO environment. It is notable that the variation in the phenotype was narrow in all of the environments. In contrast, the *FAD2-1*aabb soybean lines of population 3 containing the *FAD2-1B *I143T alleles had lower and more variable oleic acid content in the cooler environments and failed to produce a high oleic acid phenotype in either the Columbia, MO or Portageville, MO environments.

**Table 1:** Phenotype of different combinations of mutant *FAD2-1A *and mutant *FAD2-1B *produced in three environments.

Population			Oleic acid content (percent of total fatty acid)		
			
	*FAD2-1A*	*FAD2-1B*	**Costa Rica**^**1**^	**Portageville, MO**^**2**^	**Columbia, MO**^**3**^
2	Δ	P137R	81.4 ± 5.7^F5^	82.2 ± 1.2^F7^	79.1 ± 1.3^F8^
3	Δ	I143T	80.0 ± 4.0^F5^	65.0 ± 4.3^F7^	58.7 ± 7.7^F8^
4	S117N	P137R	81.1 ± 2.2^F2^	81.7 ± 2.1^F3^	77.3 ± 2.0^F3^

^1^Research station in Costa Rica. Seeds of F_5 _generation of population of 2 and 3 were produced in winter 2006-2007, while F_2 _seeds of population 4 were produced in winter 2008-2009. ^2 ^Plants were grown in Delta Research Center, seeds of F_7 _generation of the populations 2 and 3 were produced in summer 2008 and F_3 _generation of population 4 was produced in summer 2009. ^3 ^All of the plants were grown summer 2009 at the Bradford Research & Extension Center, Columbia MO

### Full fatty acid profiles and total oil and protein content

The full fatty acid profiles of the seeds of contrasting *FAD2-1 *genotypic classes produced from Populations 2, 3, and 4 in this study revealed additional alterations in palmitic acid, linoleic acid, and linolenic acid content (Table 2). As expected for a major decrease in seed expressed FAD2 enzyme activity that results in an accumulation of oleic acid, the FAD2 reaction products linoleic acid and linolenic acid were dramatically reduced in the high oleic *FAD2-1A *and *FAD2-1B *homozygous mutant lines when either of the *FAD2-1A *mutations were present along with the *FAD2-1B *P137R or I143T alleles.

**Table 2:** Fatty acid profiles for different homozygous *FAD2-1 *genotypes in four segregating populations.

Fatty Acid					
	**16:0**	**18:0**	**18:1**	**18:2**	**18:3**

Population 1 (Jake^1 ^× PI 283327)					

BB^2 ^(n = 24)	12.2 ± 0.9	3.9 ± 0.5	20.5 ± 2.6	53.4 ± 2.8	10.0 ± 0.3
bb (n = 30)	11.2 ± 0.7	3.8 ± 0.6	29.4 ± 6.0	47.0 ± 5.1	8.7 ± 0.5

Population 2 (M23 × PI283327)					

AABB (n = 5)	12.3 ± 0.5	3.7 ± 0.4	19.9 ± 3.3	55.4 ± 2.7	8.7 ± 1.0
AAbb (n = 5)	11.0 ± 0.5	3.9 ± 0.4	30.8 ± 5.2	45.9 ± 4.6	8.5 ± 0.9
aaBB (n = 14)	10.8 ± 0.8	3.8 ± 0.6	39.4 ± 5.7	37.1 ± 4.8	8.9 ± 1.2
aabb (n = 16)	7.9 ± 0.7	3.7 ± 0.6	82.2 ± 1.2	2.3 ± 0.6	3.9 ± 0.5

Population 3 (M23 × PI 567189A)					

AABB (n = 11)	12.5 ± 0.9	2.9 ± 0.4	26.3 ± 7.4	51.4 ± 6.4	6.1 ± 1.2
AAbb (n = 3)	12.4 ± 0.8	2.8 ± 0.4	31.1 ± 4.5	47.5 ± 3.3	6.1 ± 1.0
aaBB (n = 1)	10.3 ± 0.6	2.8 ± 0.3	48.2 ± 7.2	32.5 ± 6.1	6.2 ± 0.9
aabb (n = 16)	8.4 ± 0.8	2.6 ± 0.4	80.0 ± 4.0	5.0 ± 3.0	3.8 ± 0.6

Population 4 F_2_(17D × S08-14788)					

AABB (n = 5)	12.3 ± 0.9	3.2 ± 0.3	20.1 ± 0.9	55.7 ± 1.0	8.7 ± 0.6
AAbb (n = 5)	12.1 ± 1.0	3.4 ± 0.5	26.5 ± 4.5	47.8 ± 3.7	10.2 ± 0.9
aaBB (n = 6)	11.7 ± 0.3	3.0 ± 0.2	26.8 ± 1.4	48.2 ± 0.7	9.9 ± 0.5
aabb (n = 12)	7.8 ± 0.5	3.1 ± 0.2	81.1 ± 2.2	3.2 ± 1.4	4.9 ± 0.6

Population 4 F_2:3 _(17D × S08-14788)					

AABB (n = 5)	9.6 ± 0.6	3.9 ± 0.4	22.4 ± 2.9	56.0 ± 2.8	8.2 ± 0.9
AAbb (n = 4)	10.5 ± 0.5	3.8 ± 0.3	23.1 ± 2.5	54.0 ± 2.6	8.6 ± 0.5
aaBB (n = 6)	9.3 ± 0.6	3.2 ± 0.3	35.0 ± 7.8	42.9 ± 5.9	9.6 ± 2.2
aabb (n = 11)	6.9 ± 0.4	3.2 ± 0.2	77.3 ± 2.0	6.3 ± 1.5	6.3 ± 0.6

^1^Jake is a soybean line with normal oleic acid content and wild-type *FAD2-1A*; M23 and 17D are soybean lines with mutant *FAD2-1A*Δ and *FAD2-1A *S117N alleles, respectively. PI 283327 and PI 567189A are soybean lines with mutant *FAD2-1B *P137R and *FAD2-1B *I143T alleles, respectively. S08-14788 is a soybean line selected from population 2 that inherited the mutant *FAD2-1B *P137R alleles from PI 283327. ^2^AA = wild-type *FAD2-1A *alleles, aa = mutant *FAD2-1A *alleles derived from M23 or 17D, BB = wild-type *FAD2-1B *alleles, bb = mutant *FAD2-1B *alleles derived from PI 283327 or PI 567189A.

By evaluating the proportions of oleic, linoleic, and linolenic acids present in the oil extracted from mature seeds, the relative *in vivo *FAD2 and FAD3 desaturase activities of the developing seeds were estimated for the contrasting homozygous *FAD2-1 *genotypes from each population. The *FAD2-1 *AABB genotypes contained estimated FAD2 desaturase activities (the sum of the final linoleic and linolenic acid contents divided by the sum of final oleic, linoleic, and linolenic acid contents, expressed as a percent) of 76%, 76%, and 74% for Population 2, Population 3, and Population 4, respectively. The *FAD2-1 *aabb genotypes contained FAD2 desaturase activities of 7%, 10%, and 14%, for Population 2, Population 3, and Population 4, respectively. Also noted is that the accumulation of linolenic acid follows a different pattern for the *FAD2-1 *aabb mutant lines compared to the *FAD2-1 *AABB lines, with increased estimated FAD3 desaturase activity (final linolenic acid content divided by the sum of final linoleic and linolenic acid contents, expressed as a percent) for the *FAD2-1 *mutant lines.

While no significant differences were observed for the stearic acid levels in the contrasting *FAD2-1 *genotypes, the *FAD2-1 *aabb mutant lines consistently produced lower palmitic acid levels than lines with the *FAD2-1 *AABB genotype. The most dramatic change was for Population 2. In that case, the content of palmitic acid was 7.9% for the *FAD2-1 *aabb mutant lines compared to 12.3% for the *FAD2-1 *AABB lines.

Because of the concern that improvement in fatty acid profiles might have negative impacts on the total oil and protein profiles of the seeds, we also evaluated the protein and oil contents for the field produced F_2:3 _seeds from Population 4. There were no significant differences in the protein or oil contents among the different homozygous *FAD2-1 *genotypes, or with those lines compared to either Williams 82 or the 17D parental line. The *FAD2-1B *P137R allele donor parental line had a minor decrease in the average oil content and the highest mean protein content of all of the lines examined.

## Discussion and Conclusions

Increasing the oleic acid content in soybean seed oil is one of the most effective and efficient ways to enhance the nutritional value and practical utilization of soybean oil. However, the previously characterized sources of elevated oleic acid soybean involved mutation of the *FAD2-1A *gene alone, which failed to achieve oleic acid levels stable above 60% of the oil [19,21,32] or utilized approaches that have proven to have limited usage in soybean breeding due to the complexity of the trait [23]. A transgenic approach was successful in downregulation of the *FAD2-1 *genes leading to high oleic acid levels in the oil [25,26]. We have demonstrated here that an allele of the *FAD2-1B *gene containing a conserved amino acid substitution is responsible for the elevated oleic acid content in PI 283327, since soybean lines inheriting the homozygous mutant alleles have higher levels of oleic acid compared to lines inheriting wild type *FAD2-1B *alleles. The PI 567189A *FAD2-1B *allele, which contains a very rare amino acid substitution, was also predicted to have a negative impact on the enzyme activity and function.

The most significant finding of this research was that the mutant *FAD2-1B *alleles from either PI 283327 or PI 567189A not only contribute to the elevated oleic acid content in soybean seed oil of the two germplasm accessions but also enable the accumulation of oleic acid content nearly four-fold, to more than 80% of the oil, in soybean seeds when combined with different sources of a mutant *FAD2-1A *gene. Our results define the requirement of the two major contributors to the FAD2 enzyme activity present in developing soybean seeds, *FAD2-1A *and *FAD2-1B*. Other *FAD2 *genes exist in the soybean genome and some are expressed during seed development. However, when FAD2-1A and FAD2-1B are non-functional, very little FAD2 activity appeared to remain in developing seeds, as evidenced by the minor accumulation of linoleic and linolenic acid in the seed oil of the lines containing the *FAD2-1A *and *FAD2-1B *mutant combinations.

Our study demonstrated that different types of *FAD2-1A *mutant alleles bring about a similar high level of oleic acid content in the oil of soybean seeds when combined with the P137R *FAD2-1B *allele from PI 283327. M23 has lost its *FAD2-1A *alleles during the X-ray treatment while 17D carries the alleles with a mutagenesis-induced mutation that appeared to be less effective in accumulating oleic acid in the seed oil [19,21]. Hence, it was assumed that 17D would be less useful than M23 in creating a high oleic acid trait. In contrast to what we expected, the *FAD2-1A *alleles from 17D were able to combine with mutant *FAD2-1B *to produce oleic acid levels similar to those when M23 was the source of the deleted *FAD2-1A *gene, and the phenotype was stable across the different environments evaluated. We conclude that for the *FAD2-1A *gene from 17D the encoded enzyme was unable to function appropriately, so when in combination with the mutant P137R *FAD2-1B *allele, the conversion of oleic acid precursors to linoleic acid precursors was almost completely blocked. Presumably there is some compensatory difference in the activity of the functional FAD2-1B when the S117N allele of *FAD2-1A *was present compared to the situation from M23 when *FAD2-1A *is deleted.

The occurrence of soybean lines with the high oleic acid phenotype in population 4 fit a model of two independent recessive genes segregating with very little evidence for additional modifier genes, demonstrating that in this circumstance only *FAD2-1A *and *FAD2-1B *are contributing to the high oleic acid content in soybean seed oil. Based on our results, we hypothesize that any soybean line carrying a null or severely mutated *FAD2-1A *allele that is crossed with PI 283327 is likely to produce individual soybean seeds in the F_2 _progeny with at least 80% oleic acid content of the seed oil.

Although there is evidence of influence of temperature on the soybean seed oleic acid content [9,10], two of our three high oleic acid soybean genotypes proved to be capable of producing a high and stable oleic acid content in three environments. Moreover, there was no reduction in oil and protein content in the evaluated high oleic acid soybean lines. Soybean lines with the combination of *FAD2-1A *Δ and *FAD2-1B *I143T alleles from population 3 failed to produce the high oleic acid phenotype when grown in the non-tropical environments. A possible explanation is the mutation in the *FAD2-1B *allele of PI 567189 A encodes at least nominal enzyme function. This explanation is supported by the fact that the I143T substitution is in a less conserved amino acid of the FAD2 enzyme than the P137R substitution. Other than that, our high oleic acid soybean lines showed a reduction of 4% at most when they were grown in the cooler environment, with a small variation in the oleic acid content. It will be necessary to test the performance of these high oleic acid soybean lines in the main North American soybean growing locations in more northern latitudes. The mutant *FAD2-1A *and *FAD2-1B *alleles will have to be combined in soybean lines with the appropriate maturity for those experiments to be conducted. However, based on the stability of the trait that we have observed so far, any reduction of oleic acid content due to the environment is likely to be minor because very little FAD2 enzyme activity remains in developing seeds in the mutant *FAD2-1A *and *FAD2-1B *lines. An additional factor is that the end use market has not matured sufficiently to define the exact oleic acid content desired for different oil uses. Another question that should be addressed is whether the trait will affect yield or other agronomic traits. It has been reported that the transgenic soybean lines with the *FAD2-1 *genes being silenced did not show any yield drag or abnormal physiology characteristics [26].

The relative contribution of FAD2-1A and FAD2-1B in oleic acid accumulation in soybean oil could not be fully explored in this study due to the lack of a true null allele of *FAD2-1B*. Previous research has indicated that *FAD2-1B *is expressed at a higher level than *FAD2-1A*, and that FAD2-1B is more stable than FAD2-1A when expressed recombinantly in yeast [5,7,19,20]. Our sequencing results of *FAD2-1A *and *FAD2-1B *alleles from 24 PIs with elevated oleic acid content revealed that the *FAD2-1A *gene sequence is much more conserved than *FAD2-1B *(data not shown). If the assumption is made that the P137R allele of *FAD2-1B *is non-functional, then the contribution of FAD2-1A to the FAD2 desaturase activity in developing seeds appears greater than that of FAD2-1B, although the variability in the fatty acid profiles for the lines that contain functional versions of FAD2-1A or FAD2-1B obscures the contribution from each allele. If the P137R allele of *FAD2-1B *retains some activity, then it could account for only one tenth of the original FAD2 activity present (in population 2, for the aabb genotypes containing a null *FAD2-1A *compared to the AABB genotypes).

Traditional breeding has been used previously to produce soybean with up to 70% oleic acid content in the seed oil [33]. However, the phenotype is not consistent across environments, and the genetics of the trait is not very well understood, which limits the usage of these soybean lines. Practices to boost oleic acid content in soybean to more than 80% have been achieved by means of suppression of the expression level of *FAD2 *genes, and as a result, transgenic high oleic soybeans were produced [25]. Commercial release of transgenic plants still has to overcome regulatory hurdles, and production and importation of transgenic plants remain unacceptable in various countries.

Our research results have demonstrated the capacity to develop soybeans containing more than 80% oleic acid in the oil based on very simple genetic manipulation, the combination of two recessive genes. As part of this research, we developed molecular marker assays that allow the selection of the desired mutant *FAD2-1A *and *FAD2-1B *alleles, even when they are present in the heterozygous state. Molecular marker selection thus eliminates the time necessary to produce an extra generation of plants that must be screened for the fatty acid phenotype. Simple genetics combined with perfect molecular marker assays will make it possible for soybean breeders to quickly incorporate the high oleic acid trait in their breeding programs. The resulting high oleic acid soybean lines can then be efficiently developed and released as cultivars to producers. Also, because only two genes control the vast majority of the high oleic acid phenotype, other genes may be added to enhance soybean oil quality such as low linolenic acid, low allergens, or a growing list of traits involved in soybean meal quality [34]. In the short term, examination of the stability of the high oleic acid soybean lines across different environments is of particular interest. Also, the soybean lines with the high oleic acid trait should be used for development of soybean varieties with favorable agronomic traits including high yield.

In conclusion, this research demonstrates that when mutant alleles of *FAD2-1A *and *FAD2-1B *are combined together by means of traditional plant breeding, they can significantly enhance the oleic acid content of the oil, up to 80%, providing a means for the development of soybean varieties with superior oil quality.

## Methods

### Population development

Recombinant inbred lines (RIL) from population 1 (F_6 _RIL of Jake × PI 283327), 2 (F_2:6 _and F_2:7 _RIL of M23 × PI283327) and 3 (F_2:5 _and F_2:7 _RIL of M23 × PI 567189 A) were created at the same time. Three crosses were made in summer 2005 at the Delta Research Center at Portageville, MO including Jake × PI 283327, M23 × PI 283327 and M23 × PI 567189A. PI 283327 and PI 567189A are two elevated oleic acid lines with maturity group V and IV, respectively (GRIN USDA), while Jake is a conventional high yielding soybean in group V that contains a typical oleic acid content [31]. M23 was selected for elevated oleic acid after mutagenesis of the cultivar Bay [35]. In 2005 and early 2006, F_1 _seeds were advanced to the F_2 _generation in Costa Rica. Each RIL tracing to a single F_2 _plant except population 1 was also advanced in Costa Rica from 2006 to 2007 for F_5 _seeds. In 2007, a bulk of five seeds from each RIL in each population was analyzed to obtain fatty acid profile for the Costa Rica location. Population 1 was grown in Portageville, MO to produce F_7 _seeds. Population 2 was grown in Portageville, MO to produce F_6 _seeds, and then soybean RILs with more than 60% oleic acid were advanced to the F_7 _generation. In population 3, only F_5 _RILs producing more than 60% oleic acid were selected to generate F_7 _seeds at Portageville, MO in subsequent generations.

Population 4 was developed from the cross 17D × S08-14788 which was created in the summer of 2008 at Portageville, MO. S08-14788 was selected from population 1 because it carried the *FAD2-1B *P137R mutant alleles derived from PI 283327. 17D is an elevated oleic soybean line developed by mutagenesis with 35% oleic acid content [21]. True F_1 _seeds were sent to Costa Rica and F_2 _seeds were produced in the winter 2009. F_2:3 _seeds were produced in Columbia, MO and Portageville, MO during the summer 2009 growing season.

Population 5 was initiated in summer 2008 at Portageville, MO. Soybean line KB07-1#123 was crossed with soybean line #93 from population 2. Soybean line #93 (> 80% oleic acid) was genotyped to contain the *FAD2-1A *Δ alleles from M23 and the *FAD2-1B *P137R alleles derived from PI 283327. KB07-1#123 is a soybean line with the pedigree [W82 × (M23 × 10-73)]. This soybean line was selected to contain three mutant alleles affecting the fatty acid profile, including *FAD2-1A *Δ alleles from M23, and mutant *FAD3A *and *FAD3C *alleles from soybean line 10-73 [21,36]. F_1 _seeds were genotyped to confirm the heterozygosity and then advanced to obtain F_2 _seeds in summer 2009 at Bradford Research and Extension Center, Columbia MO.

In 2008, populations 1 and 2 were grown in Portageville, MO to produce the seeds analyzed for fatty acids in figures 2 and 3. In 2009, population 4 was grown in Columbia, MO to produce the seeds analyzed for fatty acid analysis in figure 5. Data in figure 4 was from F_5 _seeds of population 3 produced in Costa Rica. In addition, five lines with the highest oleic acid content from populations 2 and 3 were grown in Columbia, MO in 2009. Similarly, four to eleven lines from each of four combinations of homozygous *FAD2-1A *and *FAD2-1B *genes from population 4 were grown in Columbia MO and selected lines from population 4 were grown in Portageville, MO in 2009.

### DNA isolation and PCR for sequencing of *FAD2-1A *and *FAD2-1B*

Genomic DNA was isolated from approximately 30 mg of dry seeds using the DNeasy Plant Mini Kit (Qiagen, Inc., Valencia, CA) and used at 5 to 50 ng per PCR reaction. PCR was carried out using Ex Taq according to manufacturer's recommendation (Takara, Otsu, Shiga, Japan) in a PTC-200 thermocycler (MJ Research/Bio-Rad, Hercules, CA). Primers for *FAD2-1A: *forward was ACTGCATCGAATAATACAAGCC and reverse was TGATATTGTCCCGTGCAGC. Primers for *FAD2-1B*: forward was CCCGCTGTCCCTTTTAAACT and reverse was TTACATTATAGCCATGGATCGCTAC. PCR was programmed as the following: 95°C for 5 minutes followed by 35 cycles of 95°C for 30 seconds, 60°C for 30 seconds, and 72°C for 1 minute 30 seconds. PCR products were examined for size by running on Flashgel (Lonza Group Ltd., Switzerland) for 5 minutes. PCR products were then isolated with the Qiaprep Spin Miniprep kit (Qiagen, Inc.) and sequenced at the University of Missouri DNA Core facility.

### Sequence analysis

Sequences were aligned using Multiple Sequence Alignment by CLUSTALW http://align.genome.jp/, and evaluated for variant nucleotides between 'Williams 82' reference http://www.phytozome.net/soybean and the PIs. Protein translation was conducted using ExPaSy http://ca.expasy.org/tools/dna.html and protein alignment was done using Multiple Sequence Alignment program.

### *FAD2-1B *allele specific molecular marker assay

SimpleProbe assays were based on the disassociation kinetics of SimpleProbe obligonucleotides (Roche Applied Sciences) to be exactly complimentary to the 'Williams 82' reference sequence. SimpleProbe was purchased from Roche Applied Sciences. The *FAD2-1B *probe consists of 5'-Fuorescence-AGTCCCTTATTTCTCATGGAAAA**T**AAGC--Phosphate-3'. C > G mutation (P137R allele from PI 283327) is indicated by underline and T > C mutation (I143T allele from PI 567189A) is indicated by bold font. Primers used to generate template for Simpleprobe genotyping assay were designed by aligning the *FAD2-1A *and *FAD2-1B *region containing the SNPs. Primers were selected to be as close as possible to the SNPs while differing in at least 3 nucleotides between the two genes to specifically amplify the targeted region in *FAD2-1B*. Genotype reactions used asymmetric PCR to generate additional single stranded DNA to which the Simpleprobe could bind with less competition from the opposite amplification stand. Because the *FAD2-1B *SNPs found in the two PIs were only a few nucleotides apart, the SimpleProbe was designed to detect both of the SNPs. Genotyping reactions were performed with a 5:2 asymmetric mix of primers (5'-ACTGCATCGAATAATACAAGCC-3' at 2 μM final concentration, and 5'-TGATATTGTCCCGTCCAGC-3' at 5 μM final concentration). Reactions were carried out in 20 μl; containing template, primers, 0.2 μM final concentration of SimpleProbe, buffer (40 mM Tricine-KOH [pH 8.0] 16 mM MgCl_2_, 3.75 μg ml^-1 ^BSA,), 5% DMSO, 200 μM dNTPs, and 0.2X Titanium Taq polymerase (BD Biosciences, Palo Alto, CA). Genotyping reactions were performed using a Lightcycler 480 II real time PCR instrument (Roche), using the following PCR parameters: 95°C for 5 minutes followed by 40 cycles of 95°C for 20 seconds, 60°C for 20 seconds, 72°C for 20 seconds, and then a melting curve from 50°C to 70°C. When DNA from PI 283327 and PI 567189A is amplified with gene specific primers and used in melting curve analysis with the SimpleProbe, a mismatch between the Simpleprobe and the amplicon results in altered disassociation kinetics. Each genotype produced a characteristic melting profile, as measured by Tm of the negative first derivative of the disappearance of fluorescent signal. PI 283327 and all soybean lines with an identical *FAD2-1B *allele genotype have a characteristic peak at 56.7°C, while the PI 567189A *FAD2-1B *allele genotype yielded a characteristic peak at 60.2°C. M23 and Jake (wild-type *FAD2-1B*) have a peak at 62.5°C. Heterozygous individuals's genotype showed two peaks at either 56.7°C or 60.2°C and 62.5°C.

### *FAD2-1A *allele specific molecular marker assay for 17D

The assay was conducted as described by Dierking 2009 [21].

### *FAD2-1A *allele specific molecular marker assay for M23

An allele specific molecular marker assay was developed to distinguish soybean lines with deletion of *FAD2-1A *(*FAD2-1A *Δ alleles from M23) and the soybean lines with the presence of one (heterozygous) or two *FAD2-1A *alleles.

The reactions contain two primer pairs: one pair specific for *FAD2-1A *gene amplification and one pair specific for *phosphoenolpyruvate carboxylase 16 *(*PEPC16*) gene amplification, as an internal amplification control. The *FAD2-1A *primer pair has forward primer T2AF: (5'-ATCTTTAGATTTTTCACTACCTGGTTTAAAATTGAGGGATTG-3') and reverse primer HOLL1 (5'-CTTTGCTAGACCCTGTGTCAAAGTATAAAC-3'). The *PEPC16 *primer pair has forward primer PEPC16fwd (5'-TTCCTTTATCAGAAATAACGAGTTTAGCT-3') and reverse primer PEPC16rev (5-TGTCTCATTTTGCGGCAGC-3').

Reactions were carried out in 15 μl; each primer was at 1.3 μM final concentration in reactions containing template, buffer (40 mM Tricine-KOH (pH 8.0), 16 mM KCl, 3.5 mM MgCl_2_, 3.75 μg ml^-1 ^BSA, 200 μM dNTPs), 5% DMSO, 1.25 μM EvaGreen (Biotium Inc., Hayward, CA) and 0.2X Titanium Taq polymerase (BD Biosciences, Palo Alto, CA). PCR parameters on a DNA Engine Opticon 2 (MJ Research/Bio-Rad) were as follows: 95 μC for 5 minutes followed by 35 cycles of 95 μC for 20 seconds, 64 μC for 20 seconds, 72 μC for 20 seconds, and then a melting curve from 70 μC to 85 μC. The fluorescence was read after each cycle and every 0.2 μC with a one second hold during the melt with excitation at 470-505 nm and detection at 523-543 nm. Each genotype produced a product with a characteristic melting profile, as measured by Tm of the negative first derivative of the disappearance of fluorescent signal. Homozygous wild-type *FAD2-1A *alleles and heterozygous samples produced a peak at 76°C and possibly another peak at 78°C; homozygous mutant alleles (*FAD2-1A *Δ) only produced a peak at 78 μC. Templates for PCR were either genomic DNA samples isolated using the DNeasy Plant Mini Kit (Qiagen, Inc., Valencia, CA) or 1.2 mm washed FTA (Whatman) card punches prepared from leaves according to the manufacturer's instructions.

### Fatty acid, protein and oil determination

The method of gas chromatography of total fatty acid methyl esters of extracted oil was used to examine the fatty acid profiles of all samples [37]. The individual fatty acid contents are reported as the relative percents of palmitic, stearic, oleic, linoleic, and linolenic acids in the extracted oil. For the RIL populations, individual whole crushed seeds for each soybean line were used as samples for fatty acid determination, except the *FAD2-1*aabb lines from population 3 produced in Costa Rica, for which a bulk sample of 5 seeds was analyzed for each line. For the F_2 _seeds from population 4 and population 5, seed chips were used by removing a "chipped" portion of the seed opposite the embryo with a scalpel for fatty acid analysis such that the remainder of the seed containing the embryo could be germinated.

Protein and oil contents were determined for seeds of F_2:3 _lines for population 4 using NIR spectroscopy [38].

### Population genotyping

For every RIL population, a seed from each line was germinated in a germination package. Eight to ten days later, a small developing trifoliate leaf was excised and pressed onto an FTA card for DNA storage. Based on fatty acid profiles obtained from the other five seeds, to obtain a set of the contrasting homozygous *FAD2-1A *and *FAD2-1B *genotypes, selected lines of the three populations Jake × PI 283327, M23 × PI 283327, M23 × PI 567189A, were genotyped with all allele-specific assays as described. For the F_2 _seeds of population 4, 200 seeds were chipped, the small portion of each without hypocotyls was sent for fatty acid profiling, the remaining chipped seed was germinated to collect DNA for genotyping. Not all samples were genotyped.

## Authors' contributions

TAP and JDL contributed equally to this research. JDL and JGS selected the germplasm lines for analysis and developed the populations. TAP and KDB designed and conducted the molecular biology analyses and the association analyses. TAP and KDB drafted the manuscript with contributions and edits from JDL and JGS. All authors read and approved the final manuscript.

## Authors' information

Dr Bilyeu can also be contacted at bilyeuk@missouri.edu.
